# Neutrophil-lymphocyte ratio associated with poor prognosis in oral cancer: a retrospective study

**DOI:** 10.1186/s12885-020-07063-1

**Published:** 2020-06-17

**Authors:** Takumi Hasegawa, Tomoya Iga, Daisuke Takeda, Rika Amano, Izumi Saito, Yasumasa Kakei, Junya Kusumoto, Akira Kimoto, Akiko Sakakibara, Masaya Akashi

**Affiliations:** Department of Oral and Maxillofacial Surgery, Kobe University Graduate School of Medicine, 7-5-1, Kusunoki-cho, Chuo-ku, Kobe, 650-0017 Japan

**Keywords:** Neutrophil-to-lymphocyte ratio, Lymphocyte-to-monocyte ratio, Platelet-to-lymphocyte ratio, Oral squamous cell carcinoma, Overall survival, Disease-specific survival

## Abstract

**Background:**

Prognostic biomarkers provide essential information about a patient’s overall outcome. However, existing biomarkers are limited in terms of either sample collection, such as requiring tissue specimens, or the process, such as prolonged time for analysis. In view of the need for convenient and non-invasive prognostic biomarkers for oral cancer, we aimed to investigate the prognostic values of neutrophil-to-lymphocyte ratio, lymphocyte-to-monocyte ratio, and platelet-to-lymphocyte ratio in patient survival. We also aimed to explore the associations of these ratios with the clinicopathologic characteristics of Japanese oral squamous cell carcinoma patients.

**Methods:**

This study was a non-randomized retrospective cohort study in a tertiary referral center. We included 433 patients (246 men, 187 women) who underwent radical surgery for oral cancers between January 2001 and December 2013. We evaluated various risk factors for poor prognosis including neutrophil-to-lymphocyte ratio, lymphocyte-to-monocyte ratio, and platelet-to-lymphocyte ratio with univariate and multivariate analyses. The disease-specific survival and overall survival rates of patients were compared among the factors and biomarkers.

**Results:**

In multivariable Cox proportional hazards analysis, high neutrophil-to-lymphocyte ratio (hazard ratio 2.87, 95% confidence interval 1.59–5.19, *P* <  0.001), moderately or poorly differentiated histology (hazard ratio 2.37, 95% confidence interval 1.32–4.25, *P* <  0.001), and extranodal extension (hazard ratio 1.95, 95% confidence interval 1.13–3.35, *P* = 0.016) were independent predictors of disease-specific survival. High neutrophil-to-lymphocyte ratio (hazard ratio 2.30, 95% confidence interval 1.42–3.72, *P* <  0.001), moderately or poorly differentiated (hazard ratio 1.72, 95% confidence interval 1.07–2.76, *P* = 0.025), and extranodal extension (hazard ratio 1.79, 95% confidence interval 1.13–2.84, *P* = 0.013) were independent predictors of overall survival.

**Conclusions:**

Neutrophil-to-lymphocyte ratio might be a potential independent prognostic factor in Japanese oral squamous cell carcinoma patients.

## Background

Oral squamous cell carcinoma (OSCC) is the most common tumor of the head and neck region, and occurs predominantly in the oral cavity (90%) [1]. There have been recent improvements in the treatment of advanced OSCC. However, the survival of oral cancer patients has not dramatically improved [2]. Prognostic biomarkers are important for treatment because they provide essential information about the patients’ overall outcome. However, molecular biomarkers that need tissue specimens for analysis impose burden on patients, as it requires an invasive approach for sample collection. The analysis time for tissue biomarkers is also long. Therefore, there is an urgent need for convenient and non-invasive prognostic biomarkers for oral cancer.

It is well known that cancer has a close relationship with inflammation [3]. Inflammatory responses cause tumor progression such as initiation, progression, and metastasis [4]. The peripheral blood cell counts of the lymphocytes, monocytes, neutrophils, and platelets are reported to be associated with prognosis in several cancers [5–7]. These values can be activated by oxidative stress, chemokines, and cytokines during cancer initiation and progression [8, 9]. One report suggested that leukocytes work differently in patients with cancer and those without [10]. The neutrophil-to-lymphocyte ratio (NLR), lymphocyte-to-monocyte ratio (LMR), and platelet-to-lymphocyte ratio (PLR) are important hematological biomarkers and have been reported to be significant prognostic markers of head and neck cancer [11–13]. However, the results are controversial. Furthermore, few studies have quantitatively analyzed the usefulness of NLR, LMR, and PLR in predicting the prognosis in a small number of Japanese OSCC patients [14, 15].

In this study, we retrospectively investigated the prognostic values of NLR, LMR, and PLR in patient survival and their associations with the clinicopathologic characteristics of Japanese OSCC patients.

## Methods

This was a non-randomized retrospective cohort study. This study was approved by the institutional review board of the Kobe University Graduate School of Medicine and by the institutional review boards of the participating hospitals (Authorization number: 170086). The patient group included 433 patients (246 men, 187 women) who underwent radical surgery for OSCC between January 2001 and December 2013 at the Department of Oral and Maxillofacial Surgery, Kobe University Hospital. The mean patient age was 66.3 ± 13.5 years (range: 22–98 years). The inclusion criteria were as follows: a histological diagnosis of OSCC and the presence of a previously untreated tumor scheduled for radical surgery at initial visit. Patients with factors that could influence the NLR, LMR, and PLR such as concurrent infections, chronic inflammatory diseases, chronic hematologic diseases, and recent treatment with steroids or immunosuppressive agents were also excluded.

The data assessed for each patient included the sex, age, smoking history, alcohol consumption, performance status (PS), subsite, clinical T classification (Union Internationale Contre le Cancer/American Joint Committee on Cancer [UICC/AJCC] staging system 7th edition), clinical N classification, histological grade (well differentiated, moderately differentiated, or poorly differentiated), surgical margins, number of pathologically metastatic lymph nodes, presence of pathologic extra nodal extension (ENE), and treatment outcome. The endpoints evaluated were; the disease-specific survival (DSS) rates as the primary outcome and the overall survival (OS) rates as the secondly outcome. Survival times were calculated from the date of surgery. The peripheral NLR was calculated as the ratio of the absolute peripheral neutrophil to lymphocyte count; the peripheral LMR was calculated as the ratio of the absolute peripheral lymphocyte to monocyte count, and the peripheral PLR was calculated as the ratio of the absolute peripheral platelet to lymphocyte count. The discriminatory ability of NLR, LMR, and PLR as possible indicators of DSS was evaluated with a receiver operating characteristic (ROC) curve. This ROC curve was used to determine the cutoff values for clinical tests. The area under the resulting curve (AUC) measured the accuracy of the discrimination, ranging from 0.5 to 1. The cutoff value was chosen to minimize the number of false-positive and false-negative results. The patients were divided into two groups (the low group and high group) based on NLR, LMR, and PLR values. The DSS and OS rates of patients were compared among the patient characteristics including the NLR, LMR, and PLR.

The data were introduced into a multivariate Cox proportional hazard model in which patients were divided by age (≤ 64 years vs. ≥ 65 years), PS (0 vs. 1, 2, or 3), subsite (tongue vs. others), T stage (1 or 2 vs. 3 or 4), N stage (0 vs. others), histological grade (well vs. moderately or poorly differentiated), surgical margins (negative vs. close or positive), and number of pathologically metastatic lymph nodes (0 or 1 vs. ≥2).

### Statistical analysis

SPSS 22.0 (SPSS, Chicago, IL) and Ekuseru-Toukei 2012 (Social Survey Research Information Co., Ltd., Tokyo, Japan) were used for the statistical analyses. The association of each variable with the NLR, LMR and PLR were analyzed by the Mann-Whitney U nonparametric test for ordinal variables and the Fisher’s exact test or the Chi-squared test for categorical variables. Cumulative DSS and OS were calculated using the Kaplan–Meier product limit method. Significance among the curves was determined using the log-rank test. Probabilities of less than 0.05 were accepted as significant. All of the variables associated with the DSS or OS were introduced into multivariate Cox proportional hazard models. Hazard ratio (HR) and 95% confidence intervals (CIs) were also calculated.

## Results

The mean follow-up time among the 433 patients was 59.1 (range, 1–179) months. During the follow-up period, the 5-year OS rate and DSS were 77.3 and 84.1%. The tongue was the most common site [*n* = 211 (48.7%)].

The mean NLR, LMR, and PLR were 2.50 ± 1.73, 5.51 ± 3.47, and 143.4 ± 71.7, respectively. The optimal cutoff values of NLR, LMR, and PLR were 2.22, 4.35, and 134.3, respectively. The AUC of the NLR ROC curve was 0.72 (sensitivity, 0.71; specificity, 0.58). The AUC of the LMR ROC curve was 0.66 (sensitivity, 0.62; specificity, 0.62). The AUC of the PLR ROC curve was 0.60 (sensitivity, 0.56; specificity, 0.59).

There were significant differences between the two NLR groups in the presence of pathological multiple lymph node metastases (*P* = 0.044) in the univariate analysis (Table 1). There were no significant differences in other factors (Table 1). There were significant differences between the patients with high PS (*P* = 0.002), subsite other than the tongue (*P* = 0.015), high T stage (*P* = 0.003), and high N stage (*P* = 0.007) in the patients with low LMR in the univariate analysis (Table 2). There were no significant differences in other factors (Table 2). There were significant differences between the females (*P* <  0.001) and no smoking history (*P* = 0.002) in the high PLR group in the univariate analysis (Table 3). There were no significant differences in other factors (Table 3).

**Table 1:** Characteristics of patients according to NLR

Variables	NLR		*P* value
	Low, n (%)	High, n (%)	
Number of patients	233 (53.8)	200 (46.2)	
Sex			
Male	142 (60.9)	104 (52.0)	0.065 *
Female	91 (39.1)	96 (48.0)	
Age			
Range (Years)	27–98	22–92	
Mean ± SD	65.7 ± 13.0	67.0 ± 14.0	0.170 **
≥ 64	95 (40.8)	78 (39.0)	0.768 *
≤ 65	138 (59.2)	122 (61.0)	
Smoking history			
No	112 (48.1)	107 (53.5)	0.179 *
Yes	47 (20.2)	22 (11.0)	
Unknown	74 (31.8)	71 (35.5)	
Alcohol consumption			
No	81 (34.8)	71 (35.5)	0.472 *
Yes	76 (32.6)	55 (27.5)	
Unknown	76 (32.6)	74 (37.0)	
Performance status			
0	131 (56.2)	96 (48.0)	0.121 *
≥ 1	101 (43.4)	101 (50.5)	
unknown	1 (0.4)	3 (1.5)	
Subsite			
Tongue	116 (49.8)	95 (47.5)	0.700 *
Other	117 (50.2)	105 (52.5)	
T classification			
1, 2	167 (71.7)	134 (67.0)	0.297 *
3, 4a/b	66 (28.3)	66 (33.0)	
N classification			
0	168 (72.1)	128 (64.0)	0.078 *
Others	65 (27.9)	72 (36.0)	
Pathological status			
Pathological extranodal extensions			
ENE -	44 (57.9)	45 (54.9)	0.750 *
ENE +	32 (42.1)	37 (45.1)	
Number of pathological lymph node metastases			
0, 1	101 (69.2)	72 (57.1)	0.044 *
≥ 2	45 (30.8)	54 (42.9)	
Surgical margins			
Negative	180 (77.3)	147 (73.5)	0.358 *
Involved margins	49 (21.0)	50 (25.0)	
Unknown	4 (1.7)	3 (1.5)	
Histological differentiation			
Well differentiated	135 (57.9)	126 (63.0)	0.372 *
Moderately or poorly differentiated	95 (40.8)	73 (36.5)	
Unknown	3 (1.3)	1 (0.5)	

*ENE* Extranodal extension; *NLR* Neutrophil-lymphocyte ratio

*: Fisher’s exact test **: Mann–Whitney U test

**Table 2:** Characteristics of patients according to LMR

Variables	LMR		*P* value
	Low, n (%)	High, n (%)	
Number of patients	180 (41.6)	253 (58.4)	
Sex			
Male	112 (62.2)	134 (53.0)	0.062 *
Female	68 (37.8)	119 (47.0)	
Age			
Range (Years)	22–92	23–98	
Mean ± SD	67.8 ± 12.9	65.3 ± 13.8	0.057 **
≥ 64	66 (36.7)	107 (42.3)	0.273 *
≤ 65	114 (63.3)	146 (57.7)	
Smoking history			
No	94 (52.2)	125 (49.4)	0.680 *
Yes	32 (17.8)	37 (14.6)	
Unknown	54 (30.0)	91 (36.0)	
Alcohol consumption			
No	63 (35.0)	89 (35.2)	0.282 *
Yes	63 (35.0)	68 (26.9)	
Unknown	54 (30.0)	96 (37.9)	
Performance status			
0	78 (43.3)	149 (58.9)	0.002 *
≥ 1	100 (55.6)	102 (40.3)	
Unknown	2 (1.1)	2 (0.8)	
Subsite			
Tongue	75 (41.7)	136 (53.8)	0.015 *
Other	105 (58.3)	117 (46.2)	
T classification			
1, 2	111 (61.7)	190 (75.1)	0.003 *
3, 4a/b	69 (38.3)	63 (24.9)	
N classification			
0	110 (61.1)	186 (73.5)	0.007 *
Others	70 (38.9)	67 (26.5)	
Pathological status			
Pathological extranodal extension			
ENE -	41 (52.6)	48 (60.0)	0.423 *
ENE +	37 (47.4)	32 (40.0)	
Number of pathological lymph node metastases			
0, 1	74 (58.3)	99 (68.3)	0.101 *
≥ 2	53 (41.7)	46 (31.7)	
Surgical margins			
Negative	133 (73.9)	194 (76.7)	0.417 *
Involved margins	45 (25.0)	54 (21.3)	
Unknown	2 (1.1)	5 (2.0)	
Histological differentiation			
Well differentiated	107 (59.4)	154 (60.9)	0.736 *
Moderately or poorly differentiated	72 (40.0)	96 (37.9)	
Unknown	1 (0.6)	3 (1.2)	

ENE Extranodal extension; *LMR* Lymphocyte-to-monocyte ratio

*: Fisher’s exact test **: Mann–Whitney U test

**Table 3:** Characteristics of patients according to PLR

Variables	PLR		*P* value
	Low, n (%)	High, n (%)	
Number of patients	240 (55.4)	193 (44.6)	
Sex			
Male	156 (65.0)	90 (46.6)	< 0.001 *
Female	84 (35.0)	103 (53.4)	
Age			
Range (Years)	27–98	22–92	
Mean ± SD	66.1 ± 12.7	66.6 ± 14.4	0.253 **
≥ 64	100 (41.7)	73 (37.8)	0.431 *
≤ 65	140 (58.3)	120 (62.2)	
Smoking history			
No	115 (47.9)	104 (53.9)	0.002 *
Yes	51 (21.3)	18 (9.3)	
Unknown	74 (30.8)	71 (36.8)	
Alcohol consumption			
No	84 (35.0)	68 (35.2)	0.473 *
Yes	78 (32.5)	53 (27.5)	
Unknown	78 (32.5)	72 (37.3)	
Performance status			
0	135 (56.3)	92 (47.7)	0.081 *
≥ 1	103 (38.8)	99 (41.3)	
Unknown	2 (0.8)	2 (1.0)	
Subsite			
Tongue	126 (52.5)	85 (44.0)	0.083 *
Other	114 (47.5)	108 (56.0)	
T classification			
1, 2	176 (73.3)	125 (64.8)	0.059 *
3, 4a/b	64 (26.7)	68 (35.2)	
N classification			
0	168 (70.0)	128 (66.3)	0.467 *
Others	72 (30.0)	65 (33.7)	
Pathological status			
Pathological extranodal extension			
ENE -	49 (57.6)	40 (54.8)	0.750 *
ENE +	36 (42.4)	33 (45.2)	
Number of pathological lymph node metastases			
0, 1	94 (63.9)	79 (63.2)	0.900 *
≥ 2	53 (36.1)	46 (36.8)	
Surgical margins			
Negative	184 (76.7)	143 (74.1)	0.421 *
Involved margins	51 (21.3)	48 (24.9)	
Unknown	5 (2.1)	2 (1.0)	
Histological differentiation			
Well differentiated	142 (59.2)	119 (61.7)	0.691 *
Moderately or poorly differentiated	95 (39.6)	73 (37.8)	
Unknown	3 (1.3)	1 (0.5)	

*ENE* Extranodal extension; *PLR* Platelet-to-lymphocyte ratio

*: Fisher’s exact test **: Mann–Whitney U test

Univariate analysis showed that high T stage (*P* <  0.001), high N stage (*P* <  0.001), high NLR (*P* <  0.001), low LMR (*P* <  0.001), high PLR (*P* = 0.044), ENE (*P* = 0.004), pathological multiple lymph node metastases (*P* <  0.001), involved margins (*P* = 0.030), and moderately or poorly differentiated histology (*P* <  0.001) were associated with poor 5-year DSS (Table 4). Univariate analysis showed that high PS (*P* = 0.025), high T stage (*P* <  0.001), high N stage (*P* <  0.001), high NLR (*P* <  0.001), low LMR (*P* = 0.001), ENE (*P* = 0.012), pathological multiple lymph node metastases (*P* <  0.001), involved margins (*P* = 0.043), and moderately or poorly differentiated histology (*P* = 0.001) were associated with poor 5-year OS (Table 4).

**Table 4:** Characteristics of patients according to DSS and OS

Variables	n (%)	5-year DSS (%)	*P* value	5-year OS (%)	*P* value
Sex					
Male	246 (56.8)	83.1	0.684 *	74.6	0.126 *
Female	187 (43.2)	84.8		80.5	
Age					
Range (Years)	22–98				
Mean ± SD	66.3 ± 13.5				
≥ 64	173 (40.0)	79.9	0.120 *	73.7	0.608 *
≤ 65	260 (60.0)	86.6		79.6	
Smoking history					
No	219 (50.6)	88.2	0.548 *	82.5	0.179 *
Yes	69 (15.9)	85.3		76.7	
Unknown	145 (33.5)				
Alcohol consumption					
No	152 (35.1)	83.9	0.105 *	77.3	0.391 *
Yes	131 (30.3)	91.2		84.6	
Unknown	150 (34.6)				
Performance status					
0	227 (52.4)	85.2	0.274 *	79.6	0.025 *
≥ 1	202 (46.7)	81.9		74.3	
unknown	4 (0.9)				
Subsite					
Tongue	211 (48.7)	85.4	0.311 *	80.2	0.083 *
Other	222 (51.3)	82.2		73.9	
T classification					
1, 2	301 (69.5)	88.5	< 0.001 *	83.1	< 0.001 *
3, 4a/b	132 (30.5)	72.2		62.4	
N classification					
0	296 (68.4)	91.6	< 0.001 *	85.6	< 0.001 *
Others	137 (31.6)	66.4		58.5	
NLR					
Low (2.22 <)	233 (53.8)	91.4	< 0.001 *	84.6	< 0.001 *
High (2.22 ≥)	200 (46.2)	75.5		68.8	
LMR					
Low (4.35 <)	253 (58.4)	76.3	< 0.001 *	70.0	0.001 *
High (4.35 ≥)	180 (41.6)	89.1		82.1	
PLR					
Low (134.3 <)	240 (55.4)	86.6	0.044 *	78.4	0.232 *
High (134.3 ≥)	193 (44.6)	80.6		75.6	
Pathological status					
Pathological extra nodal metastasis					
ENE -	89 (56.3)	72.8	0.004 *	63.7	0.012 *
ENE +	69 (43.7)	50.7		42.4	
Number of pathological lymph node metastases					
0, 1	173 (63.6)	84.7	< 0.001 *	78.1	< 0.001 *
≥ 2	99 (36.4)	60.8		50.1	
Surgical margins					
Negative	314 (72.5)	85.5	0.030 *	78.7	0.043 *
Involved margins	112 (25.9)	79.1		72.1	
unknown	7 (1.6)				
Histological differentiation					
Well differentiated	261 (60.3)	91.5	< 0.001 *	83.3	0.001 *
Moderately or poorly differentiated	168 (38.8)	71.7		67.0	
unknown	4 (0.9)				

*DSS* Disease- specific survival; *ENE* Extranodal extension; *LMR* Lymphocyte-to-monocyte ratio; *NLR* Neutrophil-to-lymphocyte ratio; *OS* Overall survival; *PLR* Platelet-to-lymphocyte ratio

*: Log-rank test

**Fig. 1:**
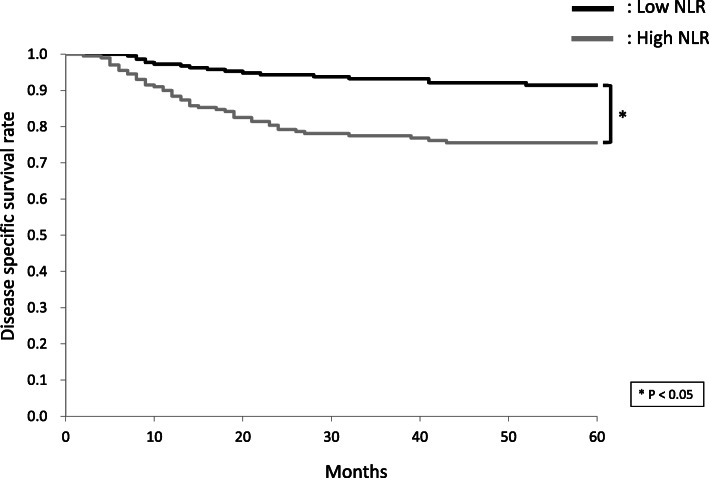
Cumulative disease-specific survival (DSS) rates in patients with high and low neutrophil-to-lymphocyte ratio (NLR). The 5-year DSS rates of patients with high and low NLR were 75.5 and 91.4%, respectively

**Fig. 2:**
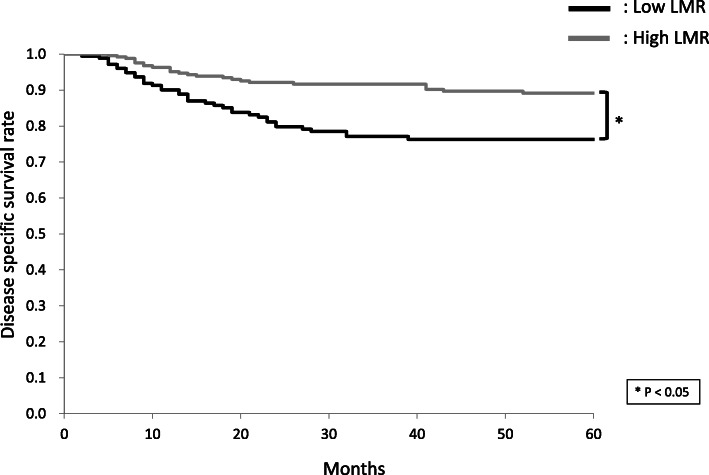
Cumulative disease-specific survival (DSS) rates in patients with high and low lymphocyte-to-monocyte ratio (LMR). The 5-year DSS rates of patients with high and low LMR were 89.1 and 76.3%, respectively

**Fig. 3:**
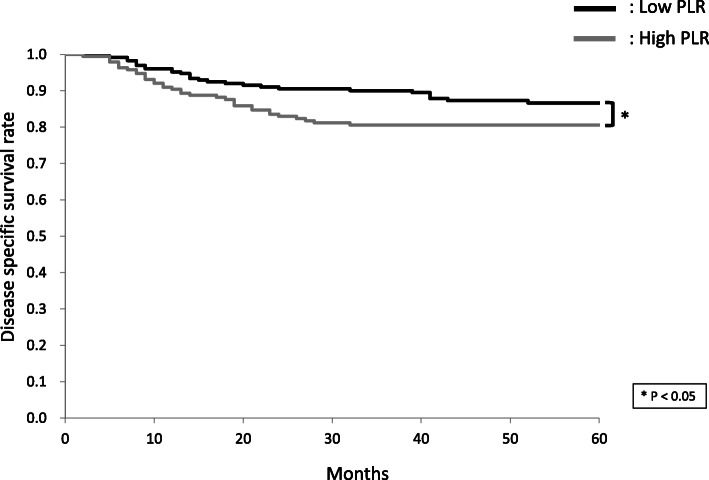
Cumulative disease-specific survival (DSS) rates in patients with high and low platelet-to-lymphocyte ratio (PLR). The 5-year DSS rates of patients with high and low PLR were 80.6 and 86.6%, respectively

The 5-year DSS rates of patients with high and low NLR were 75.5 and 91.4%, respectively (Fig. 1). The 5-year OS rates of patients with high and low NLR were 68.8 and 84.6%, respectively. The 5-year DSS rates of patients with high and low LMR were 89.1 and 76.3%, respectively (Fig. 2). The 5-year OS rates of patients with high and low LMR were 82.1 and 70.0%, respectively. The 5-year DSS rates of patients with high and low PLR were 80.6 and 86.6%, respectively (Fig. 3). The 5-year OS rates of patients with high and low PLR were 75.6 and 78.4%, respectively.

In multivariable Cox proportional hazard analysis, high NLR (Hazard ratio, HR 2.87; 95% confidence interval, CI 1.59–5.19; *P* <  0.001), moderately or poorly differentiated histology (HR 2.37, 95% CI 1.32–4.25, *P* <  0.001), and ENE (HR 1.95, 95% CI 1.13–3.35, *P* = 0.016) were independent predictors of DSS (Table 5). Also, high NLR (HR 2.30, 95% CI 1.42–3.72, *P* < 0.001), moderately or poorly differentiated (HR 1.72, 95% CI 1.07–2.76, *P* = 0.025), and ENE (HR 1.79, 95% CI 1.13–2.84, *P* = 0.013) were independent predictors of OS (Table 6).

**Table 5:** Results of multivariate Cox proportional hazards analysis of predictors of disease-specific survival

Variable	*P* value	Hazards ratio	95% CI	
			Lower	Upper
High NLR (2.22 ≥)	< 0.001	2.87	1.59	5.19
Moderately or poorly differentiated histology	< 0.001	2.37	1.32	4.25
Extranodal extension	0.016	1.95	1.13	3.35

*CI* Confidence interval; *NLR* Neutrophil-to-lymphocyte ratio

**Table 6:** Results of multivariate Cox proportional hazards analysis of predictors of overall survival (OS)

Variable	*P* value	Hazards ratio	95% CI	
			Lower	Upper
High NLR (2.22 ≥)	< 0.001	2.30	1.42	3.72
Moderately or poorly differentiated histology	0.025	1.72	1.07	2.76
Extra nodal extension	0.013	1.79	1.13	2.84

*CI* Confidence interval; *NLR* Neutrophil-to-lymphocyte ratio

## Discussion

We successfully investigated the prognostic values of NLR, LMR, and PLR in patient survival and their associations with the clinicopathologic characteristics of Japanese OSCC patients. In particular, high NLR was associated with poor prognosis.

The association between NLR and prognostic factors have been reported in various cancers [6, 16]. The measurement of NLR is very accessible and affordable because blood sampling is used. Therefore, NLR could be used as a simple indicator of systemic inflammatory responses in cancer patients. Neutrophils secrete matrix metalloproteinase 9 to promote carcinogenesis and tumor cell proliferation into the cancer microenvironment [17, 18]. In contrast, lymphocytes suppress tumor progression and are associated with an increased survival in various cancers [19, 20]. In this study, like other reports, a high NLR was associated with poor DSS and OS [15, 21, 22]. In multivariable Cox proportional hazards analysis, high NLR (HR 2.87) was an independent predictor of poor prognosis. The cutoff value of NLR ranged from 1.77 to 5 [23]. In Japan, Nakashima et al. and Sano et al. reported that the cutoff values were 2.4 and 2.36, respectively [15, 24]. In this study, the cutoff value was 2.22, like in these reports. The association between NLR and clinicopathological factors such as lymph node metastasis, T stage, differentiation, and perineural invasion was reported [23]. In this study, there were significant differences in the presence of pathological multiple lymph node metastases in the patients with high NLR like in several reports [14, 21, 22]. Therefore, NLR may be useful in predicting multiple lymph node metastasis.

The association between LMR and prognostic factors were reported in head and neck cancers [12]. Ong et al. reported that low pretreatment LMR indicated poor survival in patients with early tongue cancer [24]. In this study, low LMR was associated with poor DSS and OS like their report. A low LMR may mean a relative decrease in lymphocytes and increase in monocytes. The decreasing lymphocytes may be related to high NLR or high PLR. Tsai et al. reported that a higher pretreatment count of circulating monocytes was independently associated with poor prognosis in patients with oral cancer [25]. Chronic inflammation including cancer increases the monocyte count by the secretion of various cytokines such as TNF-α, IL-1, and IL-6 [26]. Generally, the monocytes differentiate into macrophages. Pollard et al. reported that an increased number of tumor-associated macrophages was associated with poor prognosis in cancers [27]. The cutoff value of LMR ranged from 2.35 to 5.22 [12, 24]. In this study, the cutoff value was 4.35 like these reports.

The relationship between PLR and poor prognosis is controversial. Several investigators suggested that a high PLR indicated poor prognosis in patients with head and neck SCC [13, 24]. In this study, a high PLR was associated with poor DSS. In contrast, Yu et al. indicated that preoperative PLR was not associated with survival or relapse in oral, pharyngeal, and lip cancer [28]. There are several possibilities, although the exact mechanism of the association between a high PLR and poor prognosis is not clear. Platelets can promote tumor progression by increasing angiogenesis through secretion of vascular endothelial growth factor, and invasion, and metastasis through epithelial-mesenchymal transition [13, 29–32]. The cutoff value of PLR ranged from 82 to 150 [13]. In patients with oral cancer, Chen et al. and Ong et al. reported that the cutoff values were 135 and 129 [24, 33]. In this study, the cutoff value was 134 like those reports. In multivariable Cox proportional hazards analysis of this study, a low LMR and high PLR were not independent predictors of poor prognosis. Therefore, unlike NLR and other factors, LMR and PLR may not be useful in Japanese OSCC patients.

This study had several limitations. First, this study was conducted in Japanese OSCC patients. Each cutoff value was different from other studies. Therefore, it may be clinically difficult to be used in patients of other countries. Second, the present study was retrospective and nonrandomized. Therefore, bias could not be completely excluded, although multivariate analysis was performed to decrease the effect of confounding factors as much as possible. Future research should involve a large-scale prospective cohort study to evaluate predictors of prognosis and NLR, LMR, or PLR.

## Conclusions

High NLR, moderately or poorly differentiated histology, and ENE were independent predictors of DSS and OS. In particular, high NLR was associated with poor prognosis. The NLR might be a potential independent prognostic factor in Japanese OSCC patients.

## Data Availability

All data generated or analyzed during this study are included in this published article.

## Abbreviations

AUC: Area under the curve
DSS: Disease-specific survival
LMR: Lymphocyte-to-monocyte ratio
NLR: Neutrophil-to-lymphocyte ratio
OS: Overall survival
OSCC: Oral squamous cell carcinoma
PLR: Platelet-to-lymphocyte ratio
ROC: Receiver operating characteristic curve
